# Longitudinal Extensive Myelitis After Measles in a Behçet’s Disease Patient: Post-infectious Trigger or Neuro-Behçet?

**DOI:** 10.7759/cureus.90091

**Published:** 2025-08-14

**Authors:** Amine Laabichi, Wafa Ammouri, Naima Mouatassim, Mouna Maamar, Hicham Harmouche

**Affiliations:** 1 Internal Medicine and Clinical Immunology, Hôpital Universitaire Souissi, Rabat, MAR; 2 Acute Geriatric Unit, Hôpital Universitaire Souissi, Rabat, MAR

**Keywords:** autoimmune neurology, behçet’s disease, longitudinally extensive transverse myelitis, measles, post-infectious myelitis, transverse myelitis

## Abstract

Longitudinally extensive transverse myelitis (LETM) encompasses a group of demyelinating pathologies with diverse etiologies. We report a rare case of LETM in a patient with Behçet’s disease, occurring shortly after a confirmed measles infection. This case raises a diagnostic and therapeutic dilemma between post-infectious and autoimmune causes.

A 37-year-old woman with stable Behçet’s disease developed progressive lower limb weakness two days after a measles-like exanthem. Neurological examination revealed paraparesis and a sensory level at the manubrium. Spinal MRI demonstrated a longitudinally extensive cervico-thoracic lesion with a characteristic "bagel sign." Measles serology showed positive IgM and IgG, confirming a recent primary infection. The patient was treated with intravenous methylprednisolone followed by tapering oral corticosteroids, resulting in marked clinical and radiological improvement.

This case highlights the diagnostic complexity of myelitis in patients with coexisting autoimmune conditions and recent viral infection. The favorable response to corticosteroids alone, without the use of immunosuppressive agents, supports a post-infectious etiology. This report underscores the need for individualized therapeutic decisions and the ongoing importance of maintaining high vaccination coverage.

## Introduction

Longitudinally extensive transverse myelitis (LETM) is a demyelinating condition that can result from a variety of infectious, autoimmune, or idiopathic causes. While Behçet’s disease is a known systemic vasculitis characterized by recurrent oral and genital ulcers, ocular inflammation, and vascular and joint involvement, neurological complications are less frequent. When present, they most commonly affect the diencephalon and brainstem, whereas spinal cord involvement, particularly LETM, is rare and poorly documented [1].

Measles is a highly contagious viral illness that has resurged in recent years due to declining vaccination coverage. Although neurological complications are uncommon, they may include acute encephalitis, subacute sclerosing panencephalitis (SSPE), and post-infectious demyelinating syndromes such as acute disseminated encephalomyelitis (ADEM) or transverse myelitis [2].

Since 2024, all World Health Organization (WHO) regions have reported increased numbers of measles cases, with 395,521 laboratory-confirmed measles cases reported in 2024 and 16,147 reported during the first two months of 2025 [3].

We report a case of LETM in a patient with Behçet’s disease shortly after a confirmed measles infection. This case highlights a diagnostic dilemma between post-infectious and autoimmune etiologies and raises important considerations in therapeutic decision-making.

## Case presentation

A 37-year-old woman with a seven-year history of Behçet’s disease, manifesting as bipolar aphthosis and pseudofolliculitis, treated with colchicine alone, presented with new-onset neurological symptoms. She had received only a single dose of the measles vaccine during childhood.

In late December 2024, she developed a descending erythematous rash with Koplik spots and a low-grade fever (38 °C). She was diagnosed with measles in an outpatient setting and treated symptomatically with oral vitamin A and paracetamol. Two days later, she experienced progressive heaviness in her lower limbs, prompting admission to our internal medicine department.

On examination, the patient was afebrile and in good general condition. Neurological evaluation revealed motor strength graded at 3/5 in both lower limbs (without distal predominance) and 5/5 in the upper limbs. Hypoesthesia was noted in both lower limbs, with a defined sensory level at the manubrium sternum. Deep tendon reflexes were brisk and polykinetic, and plantar reflexes were indifferent. No additional abnormalities were identified on systemic examination.

**Figure 1:**
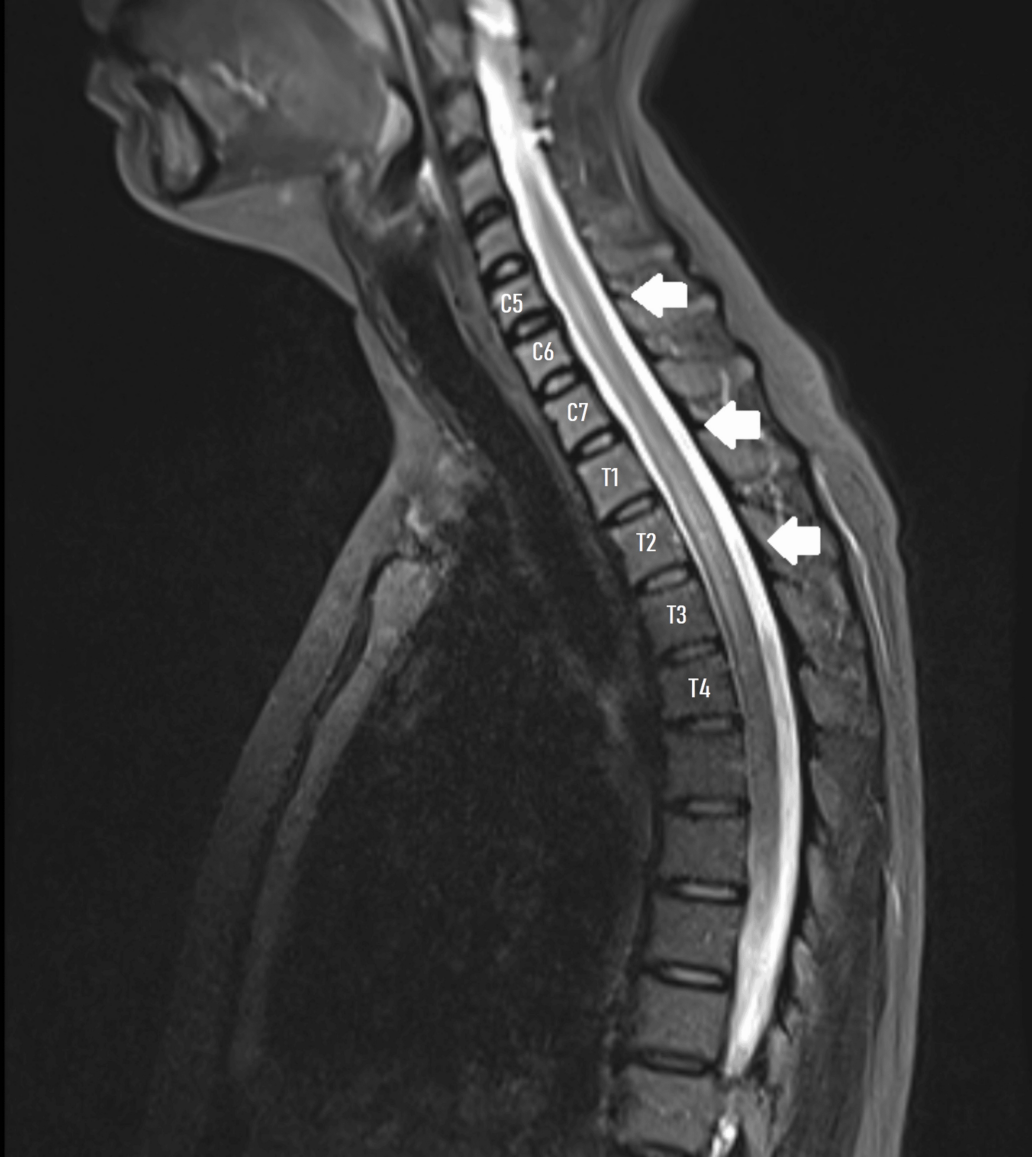
Longitudinally Extensive Spinal Cord Lesion Sagittal T2-weighted MRI of the cervical and upper thoracic spine showing a centrally located hyperintense lesion extending from C5 to T4, consistent with LETM (White Arrows), No gadolinium enhancement was seen (not shown).

**Figure 2:**
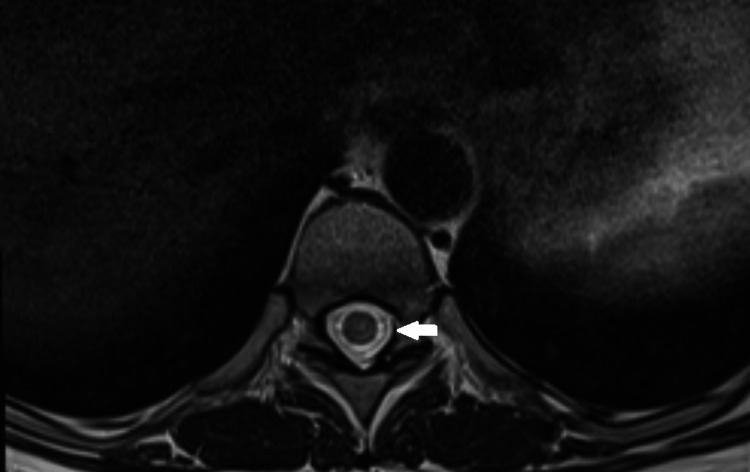
Axial MRI Showing the “Bagel Sign”. Axial T2-weighted MRI of the spinal cord demonstrating the characteristic “bagel sign” (arrow), consisting of a central T3 hypointense core surrounded by a hyperintense rim.

An urgent brain and spinal MRI-performed within 24 hours of neurological symptom onset-revealed a centrally located T2 hyperintense signal extending from C5 to T4 (Figure 1). Certain segments exhibited central T2 hypointensity, consistent with the “bagel sign” (Figure 2). No enhancement was observed after gadolinium injection. These findings were consistent with LETM. A lumbar puncture was not performed due to the patient's refusal.

Laboratory workup revealed a mild inflammatory response, with a C-reactive protein (CRP) level of 30 mg/L. Measles serology was positive for both IgM and IgG, consistent with recent primary infection. The autoimmune panel, including ANA, ANCA, anti-MOG, and anti-AQP4 antibodies, was negative. Viral serologies for hepatitis B, hepatitis C, HIV, and syphilis were also negative.

A diagnosis of LETM was made in the context of Behçet’s disease, potentially triggered by a recent measles infection. The close temporal association with the viral illness and favorable response to corticosteroids supported post-infectious myelitis. However, the longitudinal lesion extent and presence of the “bagel sign” also raised the possibility of neuro-Behçet involvement.

The patient was treated with intravenous methylprednisolone at 15 mg/kg/day for three consecutive days, followed by oral prednisone at 1 mg/kg/day with a gradual taper aiming to reach 7.5 mg/day by six months. No immunosuppressive therapy was initiated due to the rapid and sustained clinical improvement.

A dramatic clinical response was noted within days, with complete neurological recovery by three months. A follow-up spinal MRI at six months showed complete resolution of the lesions.

## Discussion

This case presents a diagnostic challenge in differentiating post-infectious longitudinally extensive transverse myelitis (LETM) from a first manifestation of neuro-Behçet’s disease. Several features favor a post-measles etiology: the close temporal relationship with a clinically and serologically confirmed measles infection, the absence of systemic Behçet’s flare, and the marked clinical and radiological improvement after corticosteroids alone, without the need for additional immunosuppressive therapy.

In contrast, certain MRI findings, notably the longitudinal lesion spanning C5-T4 and the “bagel sign” (a T2-hypointense core surrounded by hyperintensity), are more typical of neuro-Behçet’s myelitis. This sign, reported in up to 93% of myelopathic episodes in neuro-Behçet’s disease, is thought to reflect venous engorgement or acute hemorrhagic necrosis within the spinal cord [4].

In previously reported neuro-Behçet’s myelitis cases, lesions frequently extend over more than three vertebral segments and are often accompanied by brainstem or cerebral involvement in up to 60% of patients [5,6]. By contrast, post-infectious LETM (such as those following herpesviruses, Epstein-Barr virus, or SARS-CoV-2) tends to show less pronounced T2-hypointense cores and, in many cases, achieves more complete radiological and clinical resolution [7,8].

The observation that myelitis has also been documented, albeit more rarely and with milder severity, following measles vaccination [9] further supports an autoimmune mechanism for the demyelination. The present case, despite some imaging overlap with neuro-Behçet’s disease, followed a course more consistent with a monophasic post-infectious event, with full recovery and no relapses during follow-up.

Although LETM has been reported in association with various viral infections, measles-related cases remain rare [10]. Viral-associated myelitis is usually attributed to post-infectious autoimmune mechanisms, such as molecular mimicry or bystander activation, leading to demyelination. In the few published cases of post-measles LETM, onset typically occurred within weeks or months after rash resolution, and most patients responded favorably to corticosteroids, sometimes in combination with intravenous immunoglobulin (IVIG) or plasmapheresis [11]. Our patient’s presentation fits this temporal pattern; however, the co-existence of Behçet’s disease introduces an element not previously described in the literature.

To further clarify these distinctions, Table 1 summarizes the main clinical, radiological, and therapeutic differences between post-infectious LETM and neuro-Behçet’s myelitis, based on published literature [1,4-9,11].

**Table 1:** Key Clinical, Radiological, and Therapeutic Differences Between Post-infectious LETM and Neuro-Behçet’s Myelitis LETM: longitudinally extensive transverse myelitis; EBV: Epstein–Barr virus; CNS: central nervous system; CSF: cerebrospinal fluid; IVIG: intravenous immunoglobulin. Data summarized from references [1,4–9,11].

Feature	Post-infectious LETM [7,8, 11]	Neuro-Behçet’s myelitis [1,4–7]
Etiology	Immune-mediated demyelination following viral or bacterial infection (e.g., measles, herpesviruses, EBV, SARS-CoV-2)	Small-vessel vasculitis due to Behçet’s disease.
Typical onset	Days to weeks after infectious episode, rarely after a few months	Often during or after systemic Behçet’s flare.
MRI lesion length	Often ≥3 vertebral segments, may be shorter in some cases.	Often ≥3 vertebral segments, with an average of 5,5 vertebral segment .
MRI signal characteristics	T2-hyperintense without specific core.	T2-hyperintense with frequent “bagel sign” (up to 93% of cases).
Brain MRI involvement	Usually absent, a concomitant acute disseminated encephalomyelitis is described in up to 18%.	Brainstem and cerebral involvement in up to 60%.
CSF findings	Mild pleocytosis, elevated protein, negative oligoclonal bands in most.	Mild to moderate pleocytosis, occasionally neutrophil-predominant.
Course	Monophasic, with good recovery in most cases.	Relapsing–remitting or progressive; higher residual disability.
Treatment	High-dose corticosteroids ± IVIG/plasma exchange, short-term therapy.	High-dose corticosteroids plus long-term immunosuppression (azathioprine, cyclophosphamide, biologics).
Prognosis	Generally favorable, low relapse risk.	Variable, relapses common without maintenance therapy.

However, the identification of a recent viral infection should not, by itself, be taken as definitive proof of a post-infectious etiology. Other potential causes, particularly multiple sclerosis (MS) and neuromyelitis optica spectrum disorder (NMOSD), must be systematically excluded, as they require distinct, long-term immunomodulatory strategies. Inflammatory myelopathies frequently share overlapping clinical and imaging features, and premature diagnostic closure risks undertreatment or mismanagement. A thorough etiological evaluation, therefore, remains essential, especially in patients with underlying autoimmune diseases [7].

Neuro-Behçet’s disease is characterized by small-vessel vasculitis with central nervous system involvement, often affecting the brainstem and spinal cord [1]. To our knowledge, no previous reports have documented post-measles LETM in a patient with pre-existing Behçet’s disease. This unusual association raises the possibility that measles infection acted as an immune trigger in a genetically or immunologically predisposed host - through mechanisms such as molecular mimicry, bystander activation, or epitope spreading - potentially unmasking latent neuro-Behçet’s manifestations.

From a therapeutic standpoint, this case highlights the importance of initiating high-dose corticosteroids early in acute myelitis, even when the underlying cause has not yet been confirmed. While neuro-Behçet’s myelitis typically requires prolonged immunosuppression (e.g., azathioprine, cyclophosphamide, or biologics) to prevent relapses, our patient’s complete recovery without such agents argues against a chronic vasculitic process. This outcome supports an individualized, evidence-based approach in borderline cases, avoiding unnecessary exposure to the adverse effects of aggressive immunosuppressive therapy.

Finally, beyond its clinical implications, this case underscores the broader public health impact of declining measles vaccination coverage, exacerbated by increased vaccine hesitancy in the post-COVID-19 era [12]. According to the WHO, global measles incidence surged in 2024 and has continued to rise in 2025, increasing the risk of rare but severe neurological complications such as post-infectious LETM. Strengthening public confidence in routine immunization programs is therefore critical to prevent potentially devastating yet avoidable sequelae.

## Conclusions

This case illustrates the diagnostic complexity of acute LETM in patients with underlying autoimmune disease and recent viral infection. While imaging features raised suspicion for neuro-Behçet’s disease, the timing of symptoms, confirmed measles serology, and complete recovery with corticosteroids alone support a post-infectious etiology. This case reinforces the importance of early treatment, individualized immunosuppressive decision-making, and sustained vigilance in vaccination strategies to prevent potentially severe complications of preventable infections.
